# Uniclust databases of clustered and deeply annotated protein sequences and alignments

**DOI:** 10.1093/nar/gkw1081

**Published:** 2016-11-29

**Authors:** Milot Mirdita, Lars von den Driesch, Clovis Galiez, Maria J. Martin, Johannes Söding, Martin Steinegger

**Affiliations:** 1 Quantitative and Computational Biology Group, Max Planck Institute for Biophysical Chemistry, Göttingen, Germany; 2 European Molecular Biology Laboratory, European Bioinformatics Institute (EMBL-EBI), Wellcome Trust Genome Campus, Cambridge, UK; 3 Department for Bioinformatics and Computational Biology, Technische Universität München, Munich, Germany; 4 Department of Chemistry, Seoul National University, Seoul, Korea

## Abstract

We present three clustered protein sequence databases, Uniclust90, Uniclust50, Uniclust30 and three databases of multiple sequence alignments (MSAs), Uniboost10, Uniboost20 and Uniboost30, as a resource for protein sequence analysis, function prediction and sequence searches. The Uniclust databases cluster UniProtKB sequences at the level of 90%, 50% and 30% pairwise sequence identity. Uniclust90 and Uniclust50 clusters showed better consistency of functional annotation than those of UniRef90 and UniRef50, owing to an optimised clustering pipeline that runs with our MMseqs2 software for fast and sensitive protein sequence searching and clustering. Uniclust sequences are annotated with matches to Pfam, SCOP domains, and proteins in the PDB, using our HHblits homology detection tool. Due to its high sensitivity, Uniclust contains 17% more Pfam domain annotations than UniProt. Uniboost MSAs of three diversities are built by enriching the Uniclust30 MSAs with local sequence matches from MMseqs2 profile searches through Uniclust30. All databases can be downloaded from the Uniclust server at uniclust.mmseqs.com. Users can search clusters by keywords and explore their MSAs, taxonomic representation, and annotations. Uniclust is updated every two months with the new UniProt release.

## INTRODUCTION

The number of protein sequences in public databases such as UniProt (1) or GenBank (2) is growing fast, in part due to various large-scale genomics projects (3–5). The rapid growth makes it attractive for many applications to work with representative subsets, in which the representatives are computed by clustering similar sequences together and choosing only a single representative per cluster. Apart from saving computational resources, the more even coverage of sequence space of such clustered databases can improve the sensitivity of sequence similarity searches (6–8).

The popular UniProt Reference Clusters (UniRef) (9) consist of three databases that are generated by clustering the UniProtKB sequences in three steps using the CD-HIT software (10): UniRef100 combines identical UniProtKB sequences and fragments with 100% sequence identity into common entries. UniRef90 sequences are obtained by clustering UniRef100 sequences together that have at least 90% sequence identity and 80% sequence length overlap, and UniRef50 clusters together UniRef90 sequences with at least 50% sequence identity and 80% sequence length overlap.

Here, we introduce the Uniclust sequence databases which, like UniRef, are clustered, representative sets of UniProtKB sequences at three different clustering levels. But whereas UniRef relies on the CD-HIT software for the clustering, we use our software suite MMseqs2 (github.com/soedinglab/mmseqs2, Steinegger & Söding, to be published). The following characteristics make Uniclust databases unique and useful: First, the sensitivity of MMseqs2 for distantly homologous sequences allows us to cluster the UniProtKB down to 30% sequence identity. Second, we have developed a cascaded clustering workflow within MMseqs2 in order to produce sequence clusters that are as compact and functionally homogeneous as possible. As a result, Uniclust90 and Uniclust50 clusters show higher functional consistency scores than UniRef90 and UniRef50 at similar clustering depths, respectively. Third, we provide deep annotation of Uniclust sequences with Pfam (11) and SCOP (12) domains, and matches to PDB sequences (13) using HH-suite, our remote homology detection software suite. The sensitivity of HH-suite allows us to annotate 17% more Pfam domains than UniProt, which uses InterPro and HMMER3 for these annotations. Fourth, we provide the MSAs of all Uniclust clusters as well as the three Uniboost databases with MSAs of different diversity levels that are obtained by enriching Uniclust30 clusters with local sequence matches.

## MATERIALS AND METHODS

We developed an open-source bash pipeline (github.com/soedinglab/uniclust-pipeline) to generate all data described here: the Uniclust clusterings, cluster summary headers, domain annotations for sequences, and the Uniboost databases of multiple sequence alignments. We provide the pipeline scripts as a supplementary archive file to avoid cluttering the descriptions here with command line options and other details irrelevant for the understanding.

### Uniclust clustering pipeline

The Uniclust clusters contain all sequences in the UniProt knowledge base (UniProtKB), the union of the Swiss-Prot and TrEMBL databases. Sequences longer than 14 000 amino acid residues are split into multiple individual entries to limit memory usage and improve compatibility with other tools. (This affects 352 sequences in the 2016_03 release.) Once a year we will cluster these sequences from scratch as described in the following.

In order to cluster together sequences of ≥30% pairwise sequence identity, we need high sensitivity, yet the enormous number of pairwise comparisons (on the order of (10^7^)^2^) requires very high speed at the same time. We developed a cascaded clustering workflow in MMseqs (14) that uses three clustering steps with progressively increasing sensitivity and decreasing speed.

The first step consists of an extremely fast redundancy filtering that can cluster sequences of identical length and 100% overlap (‘mmseqs clusthash’). It reduces each sequence to a five-letter alphabet, computes a 64 bit CRC32 hash value for the full-length sequences, and places sequences with identical hash code that satisfy the sequence identity threshold into the same cluster. This step is run with a threshold of 90% and reduces the 61 million sequences of UniProtKB 2016_03 down to 40 million clusters in ∼20 min on a single 16-core node.

Similar to the UniRef100 clustering, we cluster fragments of sequences together with their full-length sequences. We add sequences to a cluster if they have at least 90% sequence identity to the representative sequence and are also covered by at least 95% of their length, without regard to the $E$-value.

In the first cascaded clustering step, in which we generate the Uniclust90 sequence set, we use the simple greedy clustering strategy of CD-HIT (10) that was already part of MMseqs. We assign a sequence to a cluster if it has at least 90% sequence identity with the representative sequence of the cluster and a sequence length overlap of 90% of the shorter of the two sequences. Similar to the UniRef100 clustering, to cluster fragments of sequences together with their full-length sequences we also add sequences to a cluster if they have at least 90% sequence identity to the representative sequence and are also covered by at least 95% of their length, without regard to the *E*-value.

In the third step, we generate the Uniclust50 and Uniclust30 clustering both directly from the sequences in Uniclust90, using a 50% or 30% sequence identity threshold, respectively, and a minimum sequence length overlap of 80%. A high minimum overlap ensures that all proteins within one cluster have the same or a very similar domain structure and is also an effective criterion to achieve functional homogeneity (15). We avoided the cascaded clustering approach of generating Uniclust30 from Uniclust50 because we found this resulted in slightly inferior clustering quality to the direct approach.

In addition to the simple greedy clustering, we implemented affinity propagation, depth-*n* single linkage clustering, and the classic greedy set-cover algorithm in MMseqs2 and compared the clustering qualities. We found that the cluster compactness for all algorithms could be further improved by passing over all sequences after the clustering and reassigning each to the cluster whose representative sequence is most similar to it. The greedy set-cover algorithm with sequence reassignment gave best results and is therefore used in the final clustering step. The three-step clustering took 5 days on 10 nodes with two Intel Xeon E5-2640 v3 CPUs and 128GB main memory each.

#### Updating Uniclust

We will update the Uniclust databases every two months following the new UniProt release. To keep the cluster identifiers stable between updates, we do not recluster from scratch but instead update the clustering incrementally, add new sequences to existing clusters, create new clusters, and remove deprecated sequences (14). We employ the updating workflow ‘mmseqs clusterupdate’ in the MMseqs2 package for that purpose, which has the added advantage of running in linear time instead of quadratic in the number of sequences. To avoid excessive computational demands, we recompute the MSAs and sequence annotations only during the reclustering step once per year and for major UniProt releases.

#### Consensus sequences and representative sequences

We provide two FASTA-formatted files for each of the Uniclust databases (see section Files for Download). One contains the representative sequences and the other the consensus sequences of each cluster. Consensus sequences are computed by running ‘mmseqs result2profile’. The headers of the consensus sequence summarize the annotations of the cluster's member sequences with the top five non-redundant descriptions, giving precedence to Swiss-Prot over TrEMBL annotations and a low rank to descriptions containing *hypothetical, unknown* etc.

### Uniboost MSAs

For many applications such as secondary structure prediction, more diverse MSAs produce more accurate results. Due to the stringent sequence length overlap criterion that ensures functional homogeneity of the Uniclust30 clusters, they contain only 6 sequences on average. We therefore enrich the Uniclust30 MSAs with local sequence matches to boost their diversity. We add local alignment matches through highly sensitive iterative profile-sequence homology searches using four iterations of MMseqs2 through the database of Uniclust30 consensus sequences.

The resulting MSAs are filtered to adjust the diversity: Sequences with a BLOSUM62 score per aligned residue to the consensus sequence of less than 0.0, 0.5 and 1.1 are removed from the MSAs of the Uniboost10, Uniboost20, and Uniboost30 databases, respectively. These values were heuristically found to correspond to 10%, 20% and 30% sequence identity.

### Deep domain annotations

We first annotate Uniclust30 MSAs with matches to Pfam-A, SCOP domains, and to the PDB structure database, using our remote homology detection software HHblits, which is based on pairwise comparison of profile hidden Markov models (HMMs). Hence, we compute HMMs for Uniclust30 clusters from the corresponding Uniboost10 MSAs and search the Pfam-A, SCOP, and pdb70 databases of the HH-suite (16). These profile HMM databases are automatically kept up to date by our HH-suite server (e.g. weekly for the PDB).

To avoid multiple annotations of a region with matches to the same database, the pipeline processes matches in the order of increasing *E*-value. It accepts matches as annotation if their *E*-value is <0.01 and the database match overlaps by <10% of its aligned residues with already annotated regions.

The pipeline annotates UniProt sequences by transferring annotations of Uniclust30 MSAs to their member sequences. We need to ensure that the annotation refers to a region of the member sequence that is homologous to the annotated consensus sequence of the cluster. We therefore only transfer the cluster annotation to the member sequence if the *E*-value for the subalignment *E*_subali_ is less than 0.01: $E_{\text{subali}} = E_{\text{domain}} + K * \text{length}\_\text{consensus} * e^{\lambda s_{\text{subali}} } < 0.01$. Here *E*_domain_ is the HHblits *E*-value of the domain match, *s*_subali_ is the BLOSUM62 score of the pairwise subalignment between the consensus sequence and the member sequence overlapping the database match, and the term including *s*_subali_ is an *E*-value computed with the Karlin-Altschul statistic (17).

### Webserver

To investigate specific clusters and get familiar with the information contained in the Uniclust and Uniboost databases, we have set up a web server that offers interactive features using modern web standards and framework such as the D3.js visualization toolkit (18).

The server can perform a full-text search for keywords and sequence identifiers of over a hundred biological databases linked to UniProt entries. Searches will give a list of clusters as result, each linking to a cluster page.

**Figure 1.**
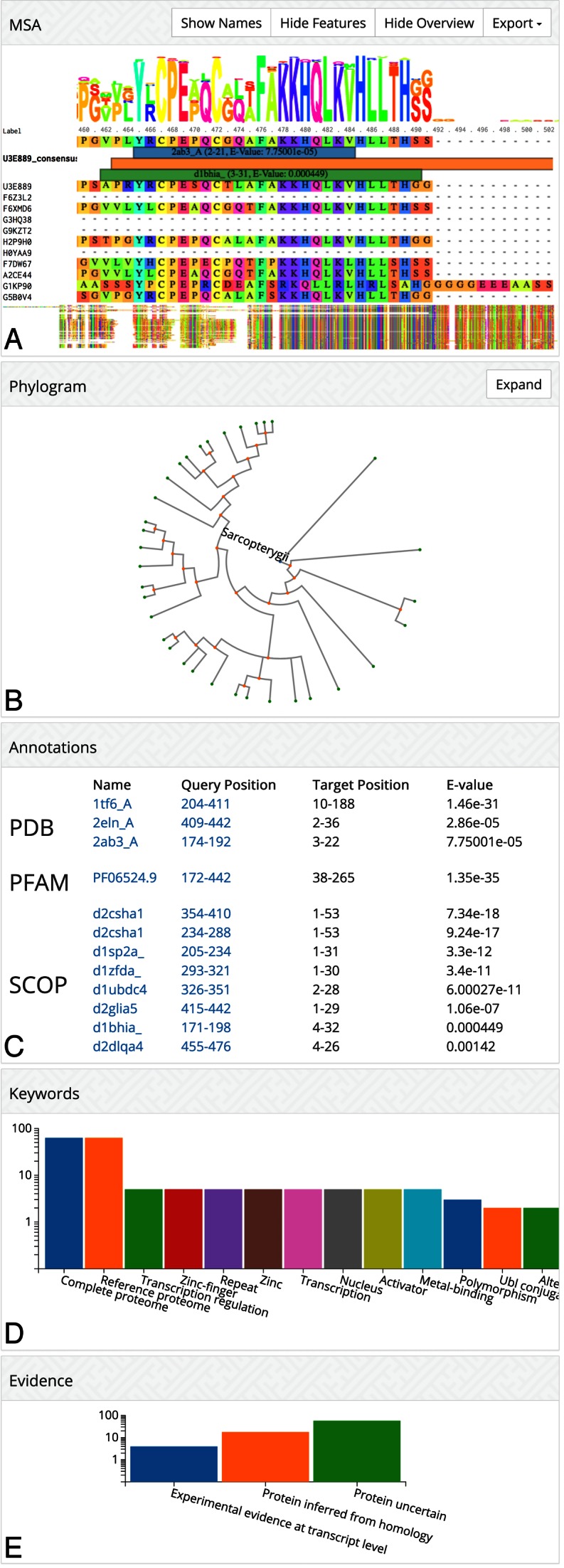
Visualization of a cluster with the multiple sequence alignment including domain annotations, the taxonomic tree for the species of the cluster's member sequences, domain annotations, summary of sequence annotation keywords, and protein evidence values.

The cluster page shows (Figure 1): (i) an interactive BioJS alignment viewer (19), which displays our Pfam, SCOP, and PDB annotations as colored bars on top; (ii) an expandable taxonomic tree (20) of the species represented in the cluster, in which the user can select sequences in the alignment viewer above; (iii) a list of annotations with links to the matched PDB, SCOP and Pfam entries; (iv) keywords occurring most frequently in the annotations; (v) a summary of protein evidence codes. Once a cluster is accessed, the URL will be stable and permanently available.

In the example of Figure 1, the keyword summary indicates that many member sequence are annotated as zinc fingers involved in transcription regulation. By following the links of the Pfam and SCOP domains they are revealed to be zinc finger domains.

### Cluster evaluation

**Figure 2.**
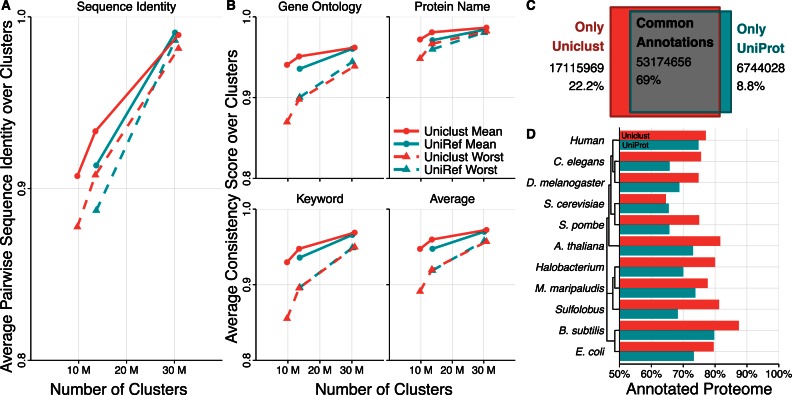
(**A**) Sequence identities averaged over all clusters of Uniclust30, Uniclust50, Uniclust90, UniRef50 and UniRef90. We compute the mean and worst sequence identity between all possible pairs of sequences in a cluster. If a cluster contains more than ten sequences we sample ten sequences to compute the sequence identities. (**B**) Annotation consistency scores averaged over all clusters of Uniclust30, Uniclust50, Uniclust90, UniRef50 and UniRef90. We compute the mean and worst annotation consistency between the representative sequence and all other cluster members for Gene Ontology annotations (top-left), protein names (top-right) keywords (bottom-left), and the average of the former three (bottom-right). (**C**) Total Pfam annotation count difference between Uniclust and UniProt. (**D**) Comparison of the fraction of proteins in ten model organisms with Pfam annotations in Uniclust and in UniProt.

In order to compare the functional homogeneity of the sequences within the same cluster, we developed scores that assess the consistency of Gene Ontology terms, keyword annotations, and protein names within the clusters. For each of these three annotation types, we defined a ‘worst’ and a ‘mean’ annotation consistency score. These are, respectively, the minimum and the mean of all pairwise annotation similarities between the representative sequence and any other sequence in the cluster. (We checked that the same results are obtained if we compare with a randomly picked sequence per cluster instead of the representative one.) This gives us 2 × 3 scores. We also compute the *average* score over the three annotation types (Figure 2B).

We now explain how the evaluation procedure computes the annotation similarities between two sequences for the three annotation types. Since there are often several Gene Ontology and keyword annotations per protein, we need similarity scores that compare the lists of annotations of two proteins.

#### Gene Ontology score

The Gene Ontology (21) is a widely used system to describe the functions of genes. It consists of three parts, to classify biological processes, cellular components in which a protein occurs, and their molecular functions. For each of these three categories, the GO annotations are organised into a hierarchical, multi-branch tree. The similarity between two Gene Ontology terms *a* and *b* is computed as proposed in (22): sim(*a, b*) = 2 log  *P*(LCA(*a, b*))/(log  *P*(*a*) + log  *P*(*b*)), where *P*(*a*) is the probability of a protein to be annotated with *a* and LCA(*a, b*) (LCA for Last Common Ancestor) is the most specific annotation node in the ontology tree that contains both *a* and *b* as child nodes.

Many proteins have multiple GO-term annotations. To obtain a GO annotation similarity value sim(*x, y*) between two proteins *x* and *y* with lists of GO annotations *A*(*x*) and *A*(*y*), we follow (23) and define the similarity between an annotation *a* of one protein with the annotation *A*_*y*_ of another, sim(*a, A*_*y*_) ≔ max {sim(*a, b*): *b* ∈ *A*_*y*_} and using it, we define the annotation similarity between proteins *x* and *y*: $\text{sim}(x,y) = ( \sum _{a \in A_x} \text{sim}(a,A_y) + \sum _{b \in A_y} \text{sim}(A_x,b) ) / ( |A_x | + | A_y | )$. Note that this similarity takes values between 0 and 1 and equals 1 if and only if *A*_*x*_ = *A*_*y*_.

#### Keyword score

Most keywords with which UniProt proteins are annotated were originally defined manually by database curators. They are automatically transferred to homologous proteins according to various rules developed within UniProt (1). The keyword annotation similarity between two proteins *x, y* with keyword lists *K*_*x*_ and *K*_*y*_ is defined in the exact same way as the GO annotation similarity while defining sim(*a, b*) = *I*(*a* = *b*), with indicator function *I*( · ). This yields sim(*x, y*) = 2|*K*_*x*_∩*K*_*y*_|/(|*K*_*x*_| + |*K*_*y*_|). The keywords in the UniProt knowledge base describe functional features in categories such as molecular function, domain, biological process, ligand and cellular component. We ignore keyword categories technical term and coding sequence diversity, and keywords provided by the UniProt automatic annotation team that do not describe biological functions.

#### Protein name score

We compute the Levenshtein string edit distance between the protein name from the ‘recommended name’ section and normalise by the length of the longer protein name to get a similarity between 0 and 1. The calculation ignores protein name entries starting with the words uncharacterized, putative, potential, probable, inactive, likely, and unknown. Additionally, we remove the uninformative word ‘protein’ from the names.

## RESULTS AND DISCUSSION

### Statistics

Table 1 shows statistics for the release 2016_03 of the Uniclust databases, which is based on the UniProt 2016_03 with 61 522 041 sequences of 325 amino acids average length.

**Table 1.** Statistics of Uniclust databases

Database	Clusters	Singletons	Average cluster size
Uniclust90	30.9 M	23.8 M	2.0 (5.4)
Uniclust50	13.5 M	9.6 M	4.6 (13.4)
Uniclust30	9.7 M	7.0 M	6.3 (19.8)

Average cluster sizes are for all clusters and, in parentheses, for non-singleton clusters.

### Clustering quality

To assess the functional homogeneity of the clusters we evaluated the mean and worst sequence identities over all clusters as measures of cluster compactness. We computed those through Clustal Omega distance matrices by running ‘clustalo --distmat-out=distance-matrix --percent-id --full --full-iter’ on all clusters. If a cluster contains more than ten sequences we sample ten random sequences for the distance matrix. Figure 2A shows these mean and worst cluster compactness values. Despite the UniRef using sequence identity and Uniclust using score-per-aligned-residue pair as similarity criterion during clustering, the Uniclust clusters have higher mean and minimum sequence identities.

Additionally to the cluster compactness we computed the annotation consistency for all clusters with respect to the Gene Ontology annotation of member sequences, the keyword consistency and the protein name consistency of each cluster's member sequences (Materials and Methods). For each annotation type we analysed the worst and the average annotation similarity between the reference sequence and the other cluster members. This analysis was performed on the Uniclust 2016_03 and UniRef 2016_03 releases based on the same version of UniProt with *N* = 61 522 041 sequences of 325 amino acids average length.

The y-axis in Figure 2B shows the consistency scores averaged over all clusters versus the number of clusters for Uniclust30, Uniclust50, Uniclust90 (red, left to right) and UniRef50, and Uniref90 (blue, left to right). Unsurprisingly, the lower the sequence identity threshold and the deeper the clustering, the fewer clusters are produced and the lower the annotation consistency scores get.

The mean scores of all annotation types show that the annotation consistencies of Uniclust90 and Uniclust50 clusters are markedly superior on average than to those of the corresponding UniRef databases.

The ‘worst’ annotation similarity per cluster is sensitive to the inclusion of even very few bad, functionally divergent sequences in the clusters. These ‘worst’ consistency scores are still quite high even for the Uniclust30, showing that the clustering produces highly pure clusters.

Note that an annotation similarity <1 between two sequences does not exclude the two sequences to have identical molecular functions but could simply be a consequence of one of the sequences being better annotated than the other. In this light, the cluster consistency scores are quite satisfactory. On the other hand, though, it is clear that many automatic annotations have been transferred on the basis of sequence similarity, which means that functional homogeneity might also be overestimated. However, such effects affect all clusterings in the same way and should therefore not invalidate the benchmark comparison. We further discuss in the supplementary material the evaluation using only GO EXP_F annotations, whose sparsity leads to a weak evaluation of the cluster consistencies.

### Annotation depth

Figure 2C compares the number of annotations of Uniclust and UniProt. Uniclust sequences contain 70 290 625 Pfam annotations, whereas UniProt sequences are annotated with 59 918 684 Pfam domains. We analysed the overlap of Uniclust and Pfam annotations by counting how many of the overlapping Uniclust and UniProt Pfam domain annotations belonged to the same Pfam family clan. On a clan level Uniclust and UniProt share 53 174 656 common annotations, while Uniclust contains 17 115 969 sequence annotations not shared by UniProt, and UniProt sequences have 6 744 028 annotations not present in Uniclust sequences.

This greater annotation depth of Uniclust is reflected in the fraction of genes with at least one Pfam domain annotation in the proteomes of various model organisms (Figure 2D). For every model organism except for *Saccharomyces cerevisiae*, Uniclust can annotate a higher percentage of the proteome.

### Availability of data

In the following we use the generic form **uniclust##_yyyy_mm.tar.gz** as placeholders for files such as **uniclust30_2016_03.tar.gz**. All downloads are available under a Creative Commons Attribution-ShareAlike 4.0 International license. We provide the following gzipped tar files for download:

- **uniclust##_yyyy_mm.tar.gz**: This archive contains three files, which will be updated every two months:
  – **uniclust##_yyyy_mm_seed.fasta**: representative (=seed) sequences of every cluster in FASTA format
  – **uniclust##_yyyy_mm_consensus.fasta**: consensus sequences of every cluster in FASTA. The sequence header starts with the Uniclust cluster identifier uc##-yymm-〈*number*〉, the UniProt accession code of the representative sequence, the size of the cluster, the up to five best functional annotations from cluster members, and UniProt identifiers of all cluster members.
  – **uniclust##_yyyy_mm_cluster_mapping.tsv**: tab-separated list with two columns of UniProt accession codes, the first for the representative sequence of the cluster, and the second for the member sequence.
- **uniboost##_yyyy_mm.tar.gz**: Uniboost database files in compressed A3M alignment format, with additional support files for HH-suite version 3.
- **uniclust30_yyyy_mm_hhsuite.tar.gz**: archive containing Uniclust multiple sequence alignments for all clusters in a3m format, generated with Clustal Omega (24), and additional support files for use with legacy HH-suite version 2 and current version 3.
- **uniclust_yyyy_mm_annotation.tar.gz**: archive containing three files with Pfam, SCOP, and PDB annotations, each formatted as tab-separated lists with nine columns: (1,2) identifiers for query and target, (3-5, 6-8) domain start and end-position and total sequence length for both UniProt and database sequence, (9) HHblits *E*-value.

## CONCLUSION AND OUTLOOK

The Uniclust databases provide functionally homogeneous clusters of sequences at three clustering depths (90%, 50% and 30% sequence identity), sets of representative sequences, MSAs of clusters, and annotations of all sequences with Pfam, SCOP, and PDB matches. The Uniclust and Uniboost MSAs are also offered as databases for HHblits, the most sensitive method for remote protein homology detection, and the provision of regular updates to these databases resolves a sore deficiency of HHblits, which was limited by very irregular and rare database updates. The MSAs in Uniboost might also prove to be a useful resource for (deep) machine-learning applications, which benefit from training on massive amounts of labeled and annotated sequence profiles.

The clustering with our MMseqs2 software currently takes around five days on 10 × 16 cores, which is sustainable for the next five to ten years due to the near-ideal scalability of MMseqs2. (The Söding lab's cluster has 640 cores at this time.) But we are also actively developing both MMseqs2 and HHblits to achieve even higher speeds and sensitivities. We expect considerable improvements in the near future in the sensitivity with which we will detect and annotate structural domains in Uniclust/UniProtKB sequences using HHblits. Similarly, extending MMseqs2 to profile-profile searches will improve the sensitivity for building the Uniboost MSAs, which again will impact the sensitivity of the domain annotations.

The Uniclust server facilitates profiting from the Uniclust databases and deep HHblits domain annotations. We hope that they will become a widely used resource for protein sequence analysis.

## Supplementary Material

Supplementary Data
