# Supplementary material for: Uniclust databases of clustered and deeply annotated protein sequences and alignments
Source: Nucleic Acids Res. 2016 Nov 29;45(Database issue):D170–6. doi: 10.1093/nar/gkw1081 (PMC5614098; doi:10.1093/nar/gkw1081)
Supplement: Supplementary Data [file gkw1081_Supp.zip › supplemental.pdf]

Supplemental: Uniclust - clustered and deeply annotated protein sequence databases

Milot Mirdita, Lars von den Driesch, Clovis Galiez,
Maria J. Martin, Johannes Söding, and Martin Steinegger

October 15th, 2016

1 Evaluation with experimentally validated annotations

As discussed in the main text, the validation may suffer from circularity, as UniProt annotations are dominantly transferred on the basis of sequence similarity. Thereby wrong homology based annotations can overestimate cluster consistency. The evaluation scores we produce can not and should not be interpreted as an absolute value of the quality of clusters, as annotations can suffer from errors and incompleteness. However the consistency scores can be used to compare different clustering algorithms or parameters of an algorithm. In our case it showed a higher consistency of Uniclust then UniRef.

Furthermore GO term associated to Uniprot entries are divided in three groups of evidence codes: experimental evidence codes and computational analysis evidence codes, which are both assigned by curators (denoted EXP_F in the sequel, with _F consisting only of functional GO annotations), and the automatically assigned evidence codes inferred from electronic annotation (IEA). In the main paper, we performed the evaluation with all evidence codes together, as the EXP_F coverage is very low and if we discard the annotations transferred by homology, there are significant disparities in the annotations depending on the type of experiment that has been carried out. As an example, Q5F3B5 and P50148 have 98.6% of sequence identity but they share a GO score of 0.0.

For sake of completeness, we evaluated the clusters of Uniclust and UniRef using only the EXP_F annotations. To cope with the disparity of the EXP_F annotations we use a procedure slightly different from the evaluation on all proteins. Specifically, to avoid strong bias depending on the choice of the representative

sequence of a cluster, we average in each cluster all against all GO score comparisons of the protein annotations falling into the EXP_F category (this also includes self-scores as the clusters have very few proteins with an EXP_F annotation, in order to be consistent with the extreme situation where one has only one EXP_F protein per cluster).

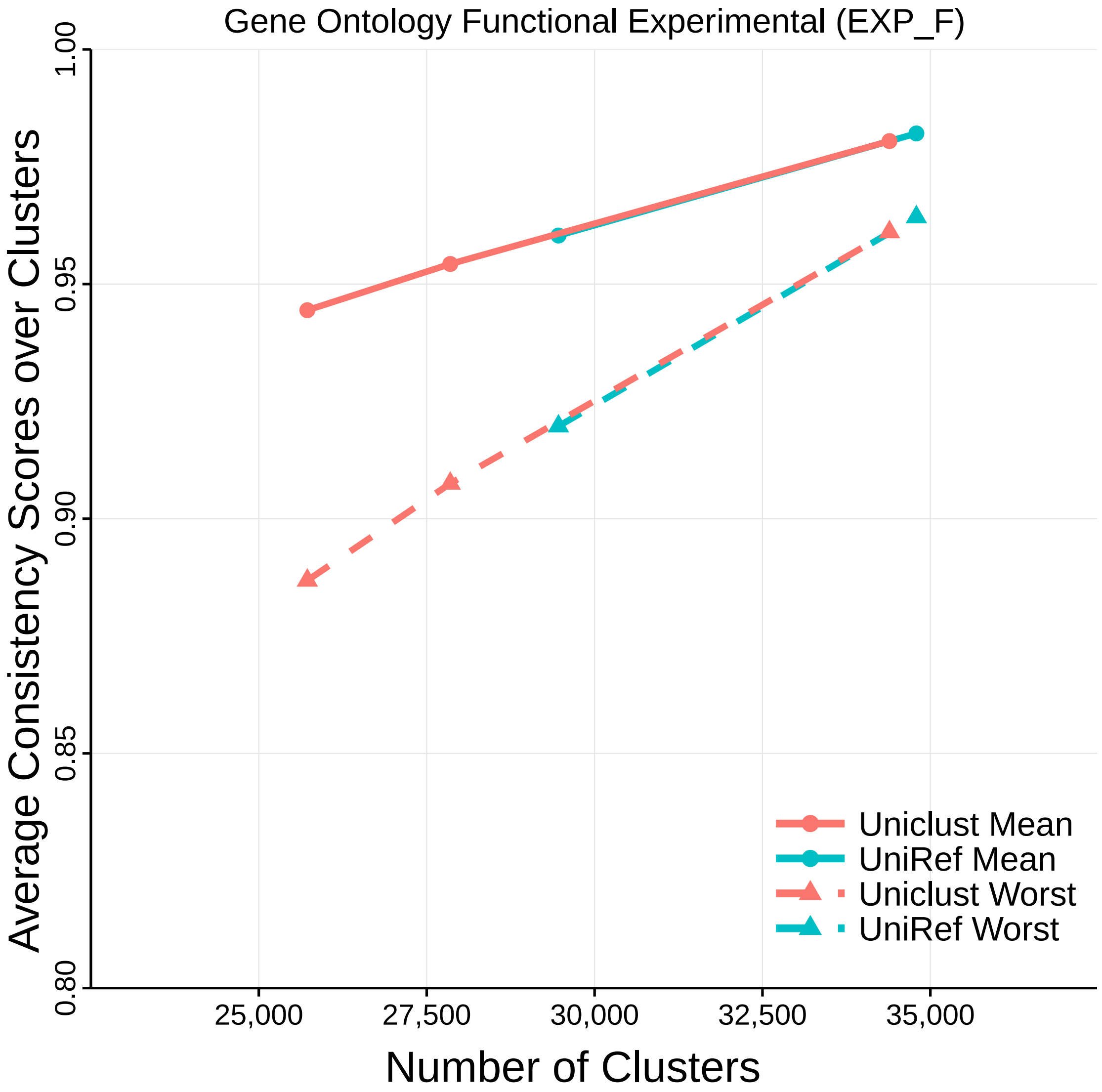

Figure 1: Evaluation with experimentally validated annotations

Note that the validation with only experimental annotation suffers from a very small coverage of clusters: only 3867 clusters in Uniclust30 include a protein with a GO EXP_F annotation.
